# Efficacy of Myofascial Techniques and Proprioceptive Neuromuscular Facilitation in the Treatment of Patients with Systemic Lupus Erythematosus—Randomized Crossover Clinical Study

**DOI:** 10.3390/healthcare13131625

**Published:** 2025-07-07

**Authors:** José-María Torres-Quiles, Rubén Cuesta-Barriuso, Raúl Pérez-Llanes

**Affiliations:** 1 Department of Physiotherapy, University of Murcia, 30120 Murcia, Spain; jm.torresquiles@um.es (J.-M.T.-Q.); rperez@um.es (R.P.-L.); 2 Department of Surgery and Medical-Surgical Specialties, University of Oviedo, 33006 Oviedo, Spain; 3 InHeFis Research Group, Instituto Asturiano de Investigación Sanitaria (ISPA), 33011 Oviedo, Spain

**Keywords:** systemic lupus erythematosus, physiotherapy, joint pain, range of motion, physical function, balance, fatigue

## Abstract

**Background/Objectives**: Systemic lupus erythematosus is an autoimmune disease. The musculoskeletal system is affected in 90% of patients. The most common symptoms are myalgia, arthralgia, and arthritis. The objective was to analyze the efficacy of an intervention using myofascial techniques and proprioceptive neuromuscular facilitation in patients with systemic lupus erythematosus. **Methods**: A randomized, single-blind, crossover clinical trial. Seventeen patients with systemic lupus erythematosus were randomly assigned to two sequences: Sequence A–B (intervention phase first, then control phase) and Sequence B–A (control phase first, then intervention phase). The intervention lasted for four weeks, with two weekly sessions lasting 50 min each. The intervention consisted of myofascial and proprioceptive neuromuscular facilitation techniques. The variables were: pain intensity (Visual Analog Scale), functional capacity of lower limbs (2-Minute Walk Test), physical function (Timed Up and Go Test), and fatigue (Fatigue Assessment Scale). After a 2-week follow-up and a 2-week washout period, the patients switched groups, and the methodology was replicated. **Results**: None of the patients developed injury or adverse effects as a direct consequence of the intervention. There were statistically significant differences between groups (*p* < 0.001) in the intensity of ankle (η^2^_p_ = 0.38) and knee (η^2^ = 0.37) pain, functional capacity (η^2^ = 0.33), and physical function (η^2^ = 0.56). There were also intergroup changes in fatigue (η^2^ = 0.52), and the relevant mental (η^2^ = 0.26) and physical (η^2^ = 0.45) components. **Conclusions**: Proprioceptive myofascial and neuromuscular facilitation techniques are safe in patients with systemic lupus erythematosus. This physical therapy protocol can improve the intensity of knee and ankle joint pain in these patients. This intervention can improve functional capacity, physical function, and fatigue in people with systemic lupus erythematosus.

## 1. Introduction

Systemic lupus erythematosus is a typical autoimmune disease, characterized by excessive activation of T and B cells, which produce a large amount of autoantibodies and proinflammatory cytokines that cause tissue and organ damage [1]. Although published data on the prevalence of systemic lupus erythematosus in Spain are scarce, it is estimated at 210:100,000 inhabitants [2]. The symptoms of this autoimmune pathology are extremely varied—from mild skin manifestations to life-threatening organ failure—so the diagnosis can be complicated, especially in the early stages [3].

Constitutional symptoms such as weight loss, fever, and feverishness are common and may be accompanied by arthralgia or arthritis. Arthritis in systemic lupus erythematosus is characterized by prolonged morning stiffness and mild to moderate joint swelling [4]. Inflammatory musculoskeletal symptoms include inflammatory arthralgia, frank sinusitis, and Jaccoud’s arthropathy, as a result of capsular laxity [5]. Skin manifestations are common and can occur in almost 80% of patients. The most frequent specific manifestation of this pathology is acute cutaneous lupus erythematosus, which can present as a butterfly rash or in the form of a generalized maculopapular rash [6].

The hematological and cardiovascular systems are involved in the development of systemic lupus erythematosus since patients with positive antiphospholipid antibodies have an increased risk of developing thrombocytopenia [7]. Pharmacological treatment of systemic lupus erythematosus includes a wide range of drugs, such as glucocorticoids, antimalarials, non-steroidal anti-inflammatory drugs, immunosuppressive agents, and biologic drugs targeting B cells [4].

Proprioceptive neuromuscular facilitation is a physiotherapy technique used to promote or accelerate the response of the neuromuscular mechanism through proprioceptor stimulation. This technique aims to promote functional movement by using facilitation, inhibition, strengthening, and relaxation [8]. The neurophysiological mechanism on which this technique is based is reciprocal innervation, postisometric relaxation (autogenous inhibition), and stress relaxation [9]. The phenomenon of autogenous inhibition leads to muscle relaxation and reduced resistance during stretching. This procedure can improve range of motion and muscle flexibility [10]. The proprioceptive receptors of myofibers and tendons are responsible for sensing the joint angle, length, and tension of muscles. Proprioceptive neuromuscular facilitation has been shown to be effective in decreasing pain, improving load balance between knee compartments, and increasing crossing speed in patients with knee osteoarthritis [11].

The myofascial system comprises the contractile muscle and connective tissue. The latter creates the shape of the muscle, penetrates into it, and orients the nerve and vascular endings. Different tissues work in harmony to form the myofascial continuum [12]. The physiological mechanism of myofascial release is to restore the length and health of restricted connective tissue to relieve pressure on pain-sensitive structures such as nerves and blood vessels [13]. A previous study in patients with systemic lupus erythematosus [14] reported how fascial release can decrease pain and increase the range of motion of the pelvic region, thoracolumbar region, and posterolateral portion of the leg, decreasing episodes of exhaustion.

The aim of this study was to analyze the efficacy of a physiotherapy intervention using myofascial techniques and proprioceptive neuromuscular facilitation in patients with systemic lupus erythematosus. We hypothesized that patients with systemic lupus erythematosus would experience greater improvements in these outcomes after the intervention phase compared to the control phase, as expected in a crossover design.

## 2. Materials and Methods

### 2.1. Study Design

Randomized, crossover, single-blind clinical study. This trial was reported in accordance with the CONSORT 2025 Statement for randomized controlled trials, including the recommendations for crossover designs, where applicable [15].

### 2.2. Participants

The patients were recruited from the Autoimmune and Lupus Association of Almería in the municipality of Nijar (Almería, Spain). This study was conducted between 12 March and 27 June 2024. For logistical reasons, the study began without prior registration of the project (this was done retrospectively at the beginning of the study), but with the prior approval of the ethics committee. The inclusion criteria of the study were: (i) subjects over 18 years of age; (ii) with a medical diagnosis of systemic lupus erythematosus; and (iii) who signed the informed consent document. On the other hand, the exclusion criteria were: (i) patients with fever or acute phlebitis; (ii) patients with recent fractures in the lower limbs; (iii) amputees; and (iv) patients with neurological or cognitive disorders that would prevent them from correctly understanding the questionnaires and performing the guided techniques.

### 2.3. Ethical Considerations

This research project was approved by the Research Ethics Committee of the San Antonio Catholic University of Murcia (code CE022409). The confidentiality and anonymity of all the data obtained were ensured, and the study complied with the ethical aspects contained in the Declaration of Helsinki of the World Medical Association. The study was registered in the international database of clinical records (NCT06383104; date: 18 April 2024).

### 2.4. Intervention

The intervention period lasted for 4 weeks, with a frequency of 2 weekly sessions of 50 min each. The intervention consisted of implementing a protocol using myofascial [16,17] and proprioceptive neuromuscular facilitation techniques [18,19].

Three low-load and a long-stretch myofascial release techniques were performed on the myofascial complex to restore optimal length, decrease pain, and improve function [20]. In the development of deep myofascial techniques, a respiratory synchronization was performed with the patient in which the physiotherapist gently placed his hands on the patient and performed 3 to 5 simultaneous breaths.

The pressure exerted by the physiotherapist was slow and progressive, respecting the adaptation of the tissue to the stimulus. The physiotherapist continued with “fascial traction” until he perceived the first restriction barrier (perceived as a stop in movement). The physiotherapist then continued to apply the stimulus until he perceived a new fascial movement, which meant that a restriction barrier had been overcome. The maneuvers used [16] by means of myofascial techniques were:

− Myofascial induction of hamstrings. This was performed with the crossed-hands technique on the sciatic tuberosity and the lower third of the posterior aspect of the thigh. The duration of the technique was from 5 to 6 min.
− Fascial induction of the triceps surae. Pressure was applied from the junction of the gastrocnemius, medially and laterally, on the internal and external gastrocnemius muscles, respectively. The thumbs acted as “control flags” of symmetry in the application of the technique. The duration of the technique was 5 min.
− Patellar myofascial release. This was applied with the cranial hand above the kneecap (lower third of the anterior rectum), exerting pressure in the cranial direction. The caudal hand was placed in the popliteal fossa, exerting traction in the caudal direction. The technique lasted 5 to 6 min.

The proprioceptive neuromuscular facilitation techniques were performed with diagonal spiral-shaped movements against resistance in a full range of motion [18]. Three sets of 3 repetitions were performed with 6-s contractions and a 30-s rest between sets [21]. The physiotherapist applied both diagonals to improve the strength and movement of a specific muscle group. Two facilitation diagonals were conducted:

− Diagonal 1. This began with a movement towards hip flexion, abduction, and internal rotation with eversion, dorsal flexion, and extension of the toes. The final action was a movement towards hip extension, adduction, and external rotation, with plantar flexion and flexion of the toes.
− Diagonal 2. The initial action was a movement towards hip abduction, extension, and internal rotation, with plantar flexion and flexion of the toes. The final action was a movement towards hip adduction, flexion, and external rotation, with dorsiflexion and extension of the toes.

Each session ended with a telescopic lower limb induction technique, where the physiotherapist gently pulled the lower limb, allowing a small external rotation thereof. After achieving three telescopic releases, adjustment movements in rotation, adduction, abduction, flexion, or extension were allowed. The physiotherapist maintained a constant traction without getting ahead of or lagging behind the patient’s movement. The intervention time was 3 to 5 min [16,17].

In the control phase, patients continued with their normal routine, without changes to their lifestyle, carrying out the activities of daily living, and with the same medical supervision as at the beginning of the study.

Myofascial techniques were applied passively when the patient received manual mechanical stimulation. On the other hand, proprioceptive neuromuscular facilitation techniques were active techniques in which the patient performed counter-resistance work. The physiotherapist who performed the intervention had experience in treating patients with systemic lupus erythematosus and holds a Master’s degree in osteopathy and manual therapy with years of clinical experience. The treatment protocol used in the intervention phase was the same for all patients, regardless of the assigned sequence order.

All participants received both conditions, but in a different order according to their assigned sequence (A–B or B–A). The detailed protocol is shown in Appendix A.

### 2.5. Measuring Instruments

The primary variable was the intensity of knee and ankle joint pain. The secondary variables were functional capacity of lower limbs and basic mobility skills, strength, balance and agility, and fatigue. Three evaluations were carried out during each phase, Phase 1 and Phase 2, of the study: before the intervention (T0), after the intervention (T1), and after a two-week follow-up period (T2). At the end of the two-week follow-up period, the patients included in the A-B sequence group were evaluated without receiving the intervention, while the patients assigned to the B-A sequence group received the intervention under the same conditions. All the evaluations were carried out by the same evaluator, blinded regarding the subject assignment to the study groups.

The intensity of knee and ankle pain was measured with the Visual Analog Scale. This instrument has an excellent test–retest reliability (ICC = 0.99) [22]. This measurement scale consists of a 10-cm line without numbering, where the patient should indicate the perceived pain in the knee and ankle joints in the last week. The range of the scale goes from 0 to 10, where 0 indicates total absence of pain.

Lower limb functional capacity was measured with the 2-Minute Walk Test (2MWT). The functional capacity for exercise, in clinical practice, was evaluated using this modified version of the 6-min version. This instrument has shown an excellent test–retest reliability (ICC = 0.97) [23]. This test was carried out in a closed 30-m-long corridor, delimited by cones. Before the test, the participants rested for at least 10 min [24]. They were allowed to use walking aids, slow down, or stop to rest if necessary [25]. The distance covered at the end of the 2 min, in meters, was recorded by the evaluator [24].

With the Timed Up and Go Test, basic mobility skills, strength, balance, and agility were assessed [26]. This instrument can help predict an individual’s risk of falls and other adverse outcomes [27]. This test has shown excellent intra-rater reliability (ICC = 0.94) [28] and is used in populations affected by diseases such as osteoarthritis [29]. This test measures the time it takes a patient to get up from a chair (without using the arms to get up), walk to a line on the floor 3 m away, turn around, and return to the chair and sit down. Two measurements were taken per patient, and the best value was used for the analysis. The unit of measurement is seconds.

Fatigue was assessed with the Fatigue Assessment Scale [30], which provides information on the physical and psychological aspects of fatigue, with a unique overall score measuring its intensity. The reliability of this scale is high (Cronbach’s alpha coefficient of 0.80) [31]. This instrument is a self-reported questionnaire consisting of 10 items with a 5-point Likert response scale. Five items reflect the physical component and five the psychological component. The scoring range of the scale is 10–50.

### 2.6. Calculation of the Sample Size and Randomization

The sample size was calculated using G*Power 3.1.9.7 software (version 3.1.9.2; Heinrich-Heine-Universität Düsseldorf, Germany), using a repeated-measures ANOVA design with interaction between intra- and inter-subject factors. This design is suitable for a crossover study with two sequence groups and six measurements per participant (three for each study period). We assumed a moderate effect size (f = 0.25), according to Cohen’s criteria, a bilateral significance level of α = 0.05, a desired statistical power of 80%, a correlation between repeated measures of 0.5, and a correction for sphericity of ε = 1. Under these assumptions, the estimated sample size was 20 participants (10 patients per sequence group). However, the study included 17 subjects, which gives a power of 99% according to the effect size obtained for the dependent variable ankle pain intensity (ŋ^2^_p_ = 0.23; f = 0.54).

The randomization was performed by a computerized randomization procedure by permuted blocks of 4 subjects in each recruitment center. The 5 possible sequence alternatives were modified in each block. The coding of the reading and assignment was performed before the inclusion of the patients, determining the order of assignment in a randomized and blinded way. This process was carried out by an assistant blinded to the assignment of groups and the identification of patients.

### 2.7. Statistical Analysis

Statistical analysis was performed using the SPSS statistical package version 19.0 for Windows (SPSS Inc., Chicago, IL, USA). The quantitative variables were described using measures of central tendency (median) and dispersion (interquartile range), while the qualitative variables were described through tables of absolute and relative frequency of the global sample.

Fisher’s exact test was used to analyze the differences in qualitative variables between the sequence groups. Normality was assessed with the Shapiro–Wilk test. The sequence, period, and carryover effects of the crossover design were evaluated using *t*-tests, given the small sample size and the structure of the data. The sequence effect was analyzed using a *t*-test for independent samples on the pre–post change in Phase 1, comparing the conditions according to the sequence of treatments (A–B vs. B–A). The period effect was evaluated using a *t*-test for related samples, comparing the clinical change between Phase 1 and Phase 2 in each subject. The carryover effect was estimated by comparing the means of the baseline clinical variable between sequences at the start of Phase 2, also using a *t*-test for independent samples. This approach has been validated and recommended for studies with small sample sizes, where methods based on mixed models may be over-adjusted or underpowered [32].

The primary analyses focused on within-subject comparisons between the intervention and control conditions, taking advantage of the crossover design, in which each participant served as their own control. A repeated-measures design was used to evaluate the effects of the intervention phase compared to the control phase, in the context of a crossover clinical trial. For each dependent variable, a repeated-measures analysis of variance (ANOVA) was applied with two within-subject factors: Condition (intervention phase vs. control phase) and Time (pre, post, follow-up), generating a 2 × 3 factorial model. In addition, the variable Sequence (A–B vs. B–A) was included as an inter-subject factor to control for possible order or carryover effects. The main effects of Time and Sequence were evaluated, as well as their interaction (Time*Sequence), which allows us to identify differences in the temporal evolution between the intervention phase and the control phase. The interactions with the Sequence variable were also explored to check for possible effects of the crossover design. In all cases, the sphericity assumption was verified using the Mauchly test; in case of violation, Greenhouse–Geisser corrections were applied. In addition, effect sizes were calculated using partial eta squared (η^2^_p_). Although linear mixed-effects models are widely recommended for the analysis of crossover clinical trials with repeated measurements, in this study, we opted for a repeated-measures ANOVA analysis for methodological and practical reasons. First, the small sample size limits the reliability of mixed models, which require a larger number of observations to accurately estimate random effects and their covariance structures. Second, the dataset was virtually complete and balanced across conditions and time points, reducing one of the key advantages of mixed models (the ability to handle missing data robustly). In addition, the experimental design, with clearly differentiated conditions by phase (intervention phase and control phase) and a counterbalanced sequence (A-B vs. B-A), is well suited to the factorial approach of repeated-measures ANOVA, allowing for clear analysis of intra- and inter-subject effects. Finally, the relevant corrections were applied (e.g., Greenhouse–Geisser in case of sphericity violation).

As a complementary analysis, the absolute pre–post intervention changes (T1–T0) were calculated in the phase in which each participant received the active treatment, to compare the results with the minimum clinically important differences (MCID). For subjects assigned to sequence A–B, Phase 1 was considered to be the intervention; in sequence B–A, Phase 2 was considered to be the intervention. Differences were calculated for the variables pain (VAS), functional capacity (2MWT), physical function (TUG), and fatigue (FAS), and were described using means and standard deviations. These changes were compared with previously published MCIDs: 1.5–2.0 points for VAS [33], 2.27 s for TUG, 14.96 m for 2MWT [23], and 4 points for FAS [34].

An intention-to-treat analysis, using baseline observation carried forward methods, was performed. According to the parameters of the sample size calculation, the statistical significance was set at *p* < 0.05 for a 95% confidence interval.

## 3. Results

### 3.1. Participants

**Figure 1:**
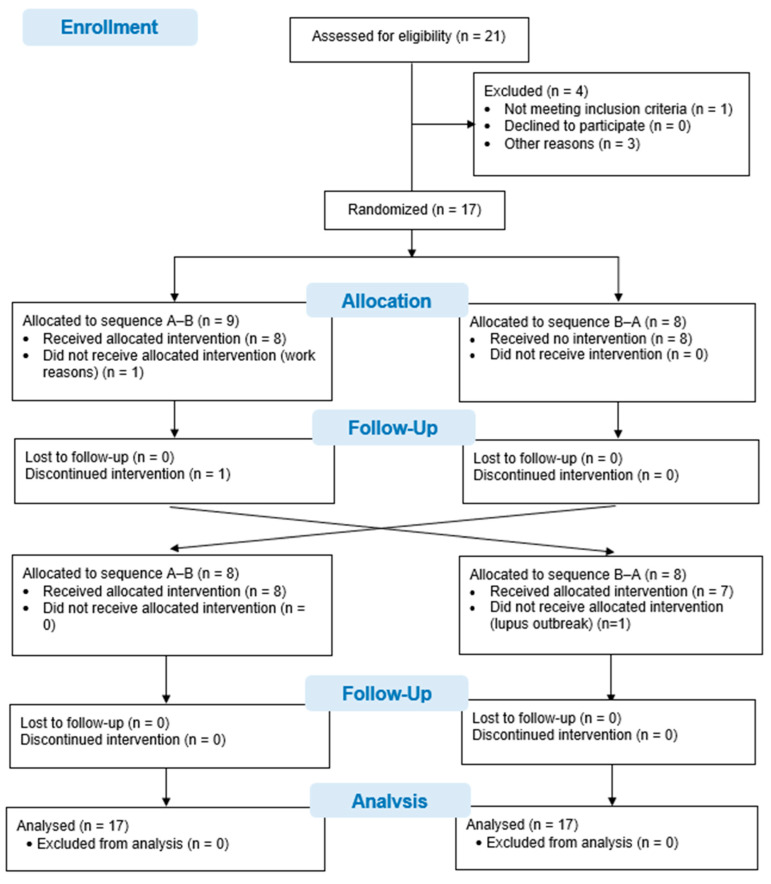
CONSORT Flow chart.

Twenty-one patients with systemic lupus erythematosus were asked to participate in this study. One patient did not meet the selection criteria when presenting with a hip fracture. Three other patients declined to participate in the study due to geographical location and time difficulties in attending the interventions and evaluations. Ultimately, 17 patients were recruited and randomized. One patient did not undergo the intervention due to difficulties with working hours, and another patient could not attend the evaluations in the second phase of the study in view of hospital admission due to an outbreak of systemic lupus erythematosus. Although 15 patients completed the study, data from all 17 patients, randomized to the 2 study sequences, were analyzed using an intention-to-treat analysis. The flow diagram for the study is shown in Figure 1.

### 3.2. Description of Sample

The mean age of the patients included in this study was 50.76 (SD: 13.18) years, with an average body mass index of 28.38 (SD: 5.85) kg/m^2^. The average number of outbreaks in the months prior to the study was 0.41 (0.71). The majority of the patients were women (82.4%) and had no family history of the disease (82.4%). The main descriptive characteristics of the sample according to the sequence group are shown in Table 1.

None of the participants who received the intervention developed any injury or adverse effects as a direct consequence of the intervention performed. Table 2 shows the main descriptive statistics of central tendency (mean) and dispersion (standard deviation) for the study variables, in the different evaluations of the two phases of this study, in each of the group sequences.

### 3.3. Analysis of the Study Effects

When comparing the clinical change in Phase 1 between the two sequences (sequence effect), statistically significant differences were found in all the study variables (*p* < 0.05), with a greater improvement in the group that received the intervention in this phase.

Analysis of the period effect did not show statistically significant differences between the changes observed in Phase 1 and Phase 2 (*p* > 0.05) in any of the study variables.

When analyzing the carryover effect, there were statistically significant differences for intensity of knee pain (*p* = 0.004; 95%CI: 1.11; 5.03) and physical function measured with the Timed Up and Go Test (*p* = 0.03; 95%CI: 0.18; 2.81). For the rest of the variables, there were no statistically significant differences (*p* > 0.05). Table 3 shows the results of the analyses of the sequence, period, and carryover effects.

### 3.4. Repeated-Measures Analysis

The apparent difference in Phase 1 (sequence effect) is attributable to the treatment effect. Similarly, no period effects were identified, and only carryover effects were observed in the variables knee pain intensity and physical function (basic mobility skills, strength, balance, and agility). Therefore, a complete repeated-measures crossover analysis was carried out.

A statistically significant effect was observed throughout the six measurements taken in this study, in all the dependent variables (*p* < 0.05). When analyzing the general differences between the intervention phase and the control phase, statistically significant differences were observed in the variables intensity of ankle pain (F_[1,15]_ = 7.24; *p* = 0.017; ŋ^2^_p_ = 0.32) and physical function, as assessed with the Timed Up and Go Test (F_[1,15]_ = 27.17; *p* < 0.001; ŋ^2^_p_ = 0.64).

In the interaction between time and the assignment sequence group, statistically significant differences (*p* < 0.001) were observed in all the study variables, with a high effect size (ŋ^2^_p_ = 0.52). Table 4 shows the results of the repeated measures analysis.

Pairwise comparisons between the intervention and control phase conditions were performed using the measurements obtained after the application of each condition (T1). This time point was selected because it reflects the immediate effect of the intervention, minimizing the influence of possible residual effects derived from the crossover design. The estimated mean differences, together with their 95% confidence intervals, allow an interpretation of the treatment effect. The results are presented in Table 5.

### 3.5. Minimal Clinically Important Differences

**Figure 2:**
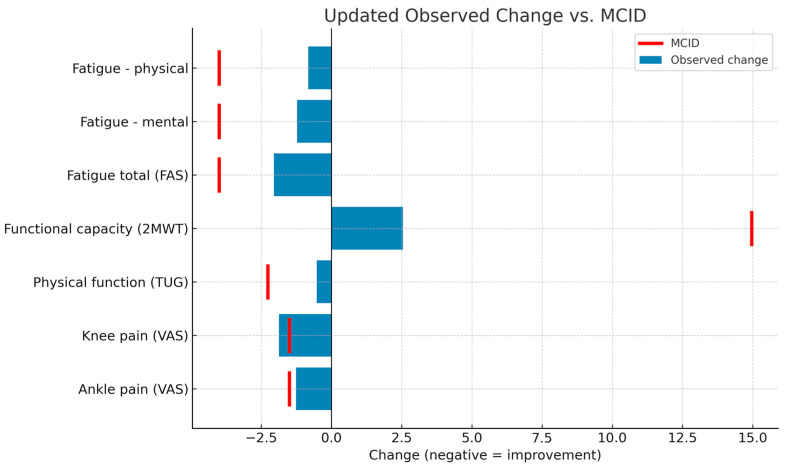
Comparison of observed intervention effects and MCID thresholds. FAS: Fatigue Assessment Scale; 2MWT: 2-Minute Walk Test; TUG: Timed Up and Go Test; VAS: Visual Analog Scale; MCID: minimum clinically important differences.

The average pre–post changes (T1–T0) during the intervention phase were calculated for each variable and compared with the minimum clinically important differences (MCID) described in the literature. The results showed that only knee pain intensity achieved a clinically relevant change (MD: −1.87 points; 95% CI: −2.92 to −0.82), exceeding the established MCID (−1.5 points). In the case of ankle pain, the mean change approached the threshold (MD: −1.26 points; MCID: −1.5 points), although it did not reach it. The remaining variables (functional capacity, physical function, and fatigue) showed mean improvements below the corresponding MCID, although in line with expected changes in short-term interventions. These findings should be interpreted in conjunction with the results of the repeated-measures analysis, which did show significant differences with moderate to large effect sizes. Figure 2 shows the changes observed against the MCID for each outcome measure.

## 4. Discussion

The aim of this study was to analyze the efficacy of a physiotherapy intervention with myofascial techniques and proprioceptive neuromuscular facilitation in improving the intensity of knee and ankle pain, physical function, and fatigue in patients with systemic lupus erythematosus. After the intervention and follow-up phase, this intervention improved the intensity of pain, physical function, and fatigue in these patients.

Musculoskeletal pain, of neuropathic, central, and mainly inflammatory origin, is a common symptom in patients with systemic lupus erythematosus [35]. In these patients, the fascia may be affected as a result of its alteration in subcutaneous, articular, and periarticular tissues, which usually begins in the fingers and progresses to the limbs. This process progresses slowly until collagen and fibrosis replace the laxer areolar tissues [36]. Myofascial techniques can modify the length and mobility of tissues by relieving pressure from pain-sensitive structures such as nerves, blood vessels, and damaged muscle tissue [13,37]. On the other hand, proprioceptive neuromuscular facilitation acts with an inhibitory effect on the Golgi tendon organs, reducing motor neuronal discharges. In this way, the musculotendinous unit relaxes by restoring its resting length and modifying the Pacini corpuscle. Therefore, proprioceptive neuromuscular facilitation could allow the relaxation of tension of the musculotendinous unit, decreasing the perception of pain in patients with systemic lupus erythematosus [38].

Patients with systemic lupus erythematosus have a lower isometric strength and atrophy of type II fibers [39], which vary according to age and sex as a result of physical inactivity and steroid therapy. We observed a significant improvement in the physical function and functional capacity of the patients. Proprioceptive neuromuscular facilitation can balance the load between the medial and lateral compartments of the knee, facilitating a reduction in pain in patients with knee osteoarthritis. Pain relief and reducing the load on the medial compartment of the knee may improve gait patterns by increasing gait and step length in older adults [11]. Similarly, muscle pain can limit physical activity and cause the formation of fibrous tissue adhesions, decreasing the range of motion. Myofascial therapy can promote a decrease in the perception of muscle pain after performing physical activity [40] and can indirectly improve the patient’s physical performance.

Fatigue is a clinical manifestation present in more than half of patients with systemic lupus erythematosus, and is associated with the course of the disease, pain, and psychosocial dysfunctions [41]. Such fatigue is related to psychosocial variables that affect patients on a daily basis, such as mood, anxiety, poor quality of sleep, and chronic pain syndromes [42]. Proprioceptive neuromuscular facilitation includes an isometric contraction of the muscle to take advantage of autogenous inhibition, which helps muscle elongation and increases muscle strength [43]. Based on this improvement in muscle strength, the perceived fatigue observed in patients with systemic lupus erythematosus included in our study may decrease. This decreased fatigue is consistent with the results observed when developing a program of progressive resistance, isometric, and stretching exercises in patients with systemic lupus erythematosus [44].

Exercise programs in patients with systemic lupus erythematosus do not complicate or affect the activity of the disease, causing beneficial effects on variables such as fatigue, depression, and cardiorespiratory capacity [45]. Resistance training has been shown to be effective in improving health-related quality of life and pain in patients with rheumatic diseases [46]. These findings coincide with the improvement in pain observed in our study after applying an intervention with proprioceptive neuromuscular facilitation and myofascial therapy.

The treatment protocol applied in the present study allows for an intervention that does not require any economic investment, and presents results in a short period of time and a safe manner. These characteristics allow equitable access for patients and the democratization of its administration, facilitating better adherence to treatment by patients.

The crossover study design requires the analysis of possible effects that may alter the results. In this study, the results of the sequence effect show different relevant effects, although this difference is consistent with the design, since in Phase 1, only one of the groups received the intervention, so it is not interpreted as a real sequence effect. In the period effect, it was observed that the timing of the treatment did not influence the clinical evolution of the patients, regardless of the intervention. Finally, the carryover effect indicates how the washout period was adequate to eliminate residual effects of the previous treatment in all variables, except for knee pain intensity and physical function (basic mobility skills, strength, balance, and agility). Regarding the internal validity of the crossover design, a baseline imbalance was detected in Phase 2 for the variables knee pain and physical function. This finding reflects a possible carryover effect of the treatment applied in Phase 1, despite a two-week washout period. Although most variables did not show significant differences at the start of the second period, this result suggests that, for certain functional measures, residual effects may persist beyond the planned washout period. In future studies, it is recommended to extend the washout period or consider parallel designs when using physiotherapeutic interventions with cumulative or long-lasting effects.

Although only one of the variables (knee pain) exceeded the minimum clinically important change (MCID) threshold, and others, such as ankle pain, approached this value, the results may have relevant clinical implications. In populations with chronic diseases such as systemic lupus erythematosus, even moderate improvements in pain, fatigue, or physical function can represent a significant advance in quality of life, especially when achieved with safe, low-cost, non-pharmacological interventions. In addition, the relatively short duration of the intervention may have limited the magnitude of change, so it is possible that a greater clinical effect may be evident with prolonged or repeated treatments. These findings, although modest, support the potential utility of the treatment as part of a multimodal approach to symptomatic management in patients with systemic lupus erythematosus.

Significant within-subject effects between the intervention phase and control phase were observed for key outcomes, as demonstrated by the repeated-measures analysis. These findings are particularly robust given the crossover design, where each participant served as their own control, thereby minimizing interindividual variability. The observed differences suggest that the intervention had a consistent and measurable impact across several clinically relevant domains. This intra-subject comparison enhances the internal validity of this study and reinforces the potential clinical value of the intervention.

The changes observed in fatigue and physical function may be partly explained by pathophysiological mechanisms specific to systemic lupus erythematosus. Central sensitization has been shown to predict fatigue independently of pain levels, underscoring the need to evaluate interventions that may reduce this phenomenon [47]. Central sensitization is characterized by heightened sensitivity to pain and fatigue due to altered central nervous system processing and may contribute to persistent symptoms even during periods of low disease activity. Additionally, fatigue in SLE is strongly influenced by chronic immune activation, proinflammatory cytokines, and neuroendocrine dysregulation [48]. The observed benefits of the intervention could reflect modulation of these mechanisms, which affect neuroimmune and psychobehavioral pathways. This perspective reinforces the relevance of non-pharmacological interventions in managing symptoms that are often refractory to immunosuppressive therapies.

### Study Limitations

The results of this study should be taken with caution due to several limitations. First of all, the required sample size was not reached in the preliminary analysis. In addition, the dropouts observed during the study may limit its power, although we attempted to correct this limitation by performing an intention-to-treat analysis.

Another limitation that should be considered is the baseline imbalance observed at the start of Phase 2 in the variables knee pain and physical function, which could indicate a carryover effect that was insufficiently controlled by the washout period. Although the statistical analysis considered sequence, period, and carryover effects, as well as the interaction between time and sequence, this finding suggests the need to strengthen the design in subsequent studies. It would be advisable to extend the washout period or apply a parallel design if it is anticipated that the effects of treatment could extend over time.

Another limitation of this study was the heterogeneity observed in the patients regarding their disease status and understanding of it, although baseline differences between groups are balanced by the crossover design, in which both arms receive the same treatment. The evaluation of variables such as perceived quality of life or anxiety would have made it possible to identify the relationship between the physical changes observed and these psychosocial perceptions that are so common in patients with systemic lupus erythematosus. Another limitation that must be considered is the low number of sessions carried out in the intervention. Increasing the washing period would be necessary to confirm that the residual effects of the previous treatment are eliminated in all the study variables. Despite the results obtained in the effects for period, sequence, and carryover, and in the Time*Sequence Interaction, with a high effect size, the low sample size of the study requires the results to be taken with caution. Future randomized clinical studies with a larger sample size should confirm the results of this study.

An additional limitation of this study is that participants were not blinded to the intervention received, and there was no sequence of patients receiving some type of sham treatment. This may have introduced a placebo or expectation effect, especially in subjective variables such as perceived pain intensity and fatigue. Although a crossover design was used, so that all the participants acted as their own control, it cannot be ruled out that the perception of receiving active treatment influenced the patients’ response. In this regard, future trials should consider including a sham intervention control condition to better discriminate between real physiological effects and those attributable to non-specific or contextual factors.

## 5. Conclusions

Proprioceptive myofascial and neuromuscular facilitation techniques are safe in patients with systemic lupus erythematosus. This physical therapy protocol can improve the intensity of knee and ankle joint pain in these patients. This intervention can improve functional capacity, physical function, and fatigue in people with systemic lupus erythematosus.

## Figures and Tables

**Table 1:** Descriptive analysis, mean (standard deviation), of the patients included in the study in the Phase 1 pretreatment assessment.

Variables	Total (n = 17)	A-B Sequence (n = 9)	B-A Sequence (n = 8)
Age (years)	50.76 (13.18)	45.56 (11.85)	56.63 (12.76)
Weight (kg)	78.26 (19.31)	79.33 (25.14)	77.06 (11.24)
Height (m)	1.65 (0.07)	1.64 (0.08)	1.66 (0.05)
Body mass index (kg/m^2^)	28.38 (5.85)	29.05 (7.74)	27.63 (2.90)
Outbreaks in the previous 6 months (number)	0.41 (0.71)	0.22 (0.44)	0.62 (0.91)
	**n (%)**		
Gender Female	14 (82.4)	8 (88.9)	6 (75.0)
Male	3 (17.6)	1 (11.1)	2 (25.0)
History of illness Yes	3 (17.6)	2 (22.2)	1 (12.5)
No	14 (82.4)	7 (77.8)	7 (87.5)

A–B: sequence intervention phase—control phase; B–A: sequence control phase—intervention phase; n: number of subjects, %: percentage.

**Table 2:** Statistics of central tendency, median, and dispersion [interquartile range] of the study variables, in both group sequences and conditions of the study.

Variable	Condition (Sequence)	T0	T1	T2
Ankle pain (0–10)	Intervention phase (sequence A-B)	4.80 [5.75]	1.80 [2.25]	1.40 [3.10]
	Intervention phase (sequence B-A)	5.00 [3.30]	1.55 [1.92]	2.05 [4.10]
	Control phase (sequence A-B)	3.00 [4.65]	4.20 [5.80]	2.00 [5.85]
	Control phase (sequence B-A)	5.95 [5.30]	5.85 [3.37]	6.65 [3.32]
Knee pain (0–10)	Intervention phase (sequence A-B)	4.30 [3.65]	1.80 [2.55]	1.40 [3.80]
	Intervention phase (sequence B-A)	7.20 [2.42]	1.60 [3.42]	1.90 [3.32]
	Control phase (sequence A-B)	3.70 [3.55]	2.90 [3.45]	3.70 [5.15]
	Control phase (sequence B-A)	8.15 [5.37]	7.05 [2.92]	6.35 [3.72]
Functional capacity of lower limbs (m)	Intervention phase (sequence A-B)	151.70 [32.18]	156.63 [27.98]	164.30 [32.15]
	Intervention phase (sequence B-A)	149.28 [46.57]	154.35 [47.88]	156.96 [43.97]
	Control phase (sequence A-B)	159.40 [38.51]	147.15 [44.58]	159.64 [44.95]
	Control phase (sequence B-A)	145.62 [50.89]	148.49 [43.76]	147.00 [52.25]
Physical function (seg)	Intervention phase (sequence A-B)	7.02 [2.15]	6.15 [1.28]	5.81 [1.97]
	Intervention phase (sequence B-A)	7.24 [2.43]	6.62 [1.89]	6.51 [1.67]
	Control phase (sequence A-B)	6.11 [1.92]	6.53 [1.39]	6.07 [2.02]
	Control phase (sequence B-A)	6.93 [2.32]	7.26 [2.61]	7.40 [2.70]
Fatigue (10–50)	Intervention phase (sequence A-B)	33.00 [6.50]	26.00 [10.50]	24.00 [10.00]
	Intervention phase (sequence B-A)	31.00 [4.75]	24.50 [6.50]	24.00 [7.00]
	Control phase (sequence A-B)	26.00 [11.50]	28.00 [7.00]	33.00 [14.00]
	Control phase (sequence B-A)	32.00 [5.00]	32.50 [3.25]	33.00 [4.25]
Fatigue-mental component (10–50)	Intervention phase (sequence A-B)	15.00 [4.00]	11.00 [4.00]	12.00 [4.50]
	Intervention phase (sequence B-A)	13.50 [1.75]	11.00 [3.50]	11.00 [2.75]
	Control phase (sequence A-B)	13.00 [4.00]	12.00 [4.50]	14.00 [6.50]
	Control phase (sequence B-A)	14.50 [2.75]	14.50 [2.00]	15.50 [2.75]
Fatigue-physical component (10–50)	Intervention phase (sequence A-B)	18.00 [4.00]	14.00 [7.00]	14.00 [6.00]
	Intervention phase (sequence B-A)	17.00 [3.50]	13.00 [2.00]	12.50 [4.75]
	Control phase (sequence A-B)	15.00 [6.00]	16.00 [5.50]	18.00 [7.50]
	Control phase (sequence B-A)	17.50 [4.25]	28.00 [2.50]	17.50 [2.00]

B–A: sequence control phase—intervention phase; A–B: sequence intervention phase—control phase; T0: outcome measures at baseline; T1: outcome measures at post-treatment assessment; T2: outcome measures at follow-up assessment.

**Table 3:** Results of the sequence, period, and carryover effect analyses.

Variables	Sequence Effect	Period Effect		Carryover Effect	
	MD [95%CI]	t	M [95%CI]	t	MD [95%CI]
Ankle pain	3.14 [0.91; 5.37] *	−0.10	−0.11 [−2.40; 2.18]	1.79	2.33 [−0.44; 5.11]
Knee pain	3.59 [1.76; 5.41] *	0.65	0.71 [−1.61; 3.05]	3.48	3.07 [1.11; 5.03] *
Functional capacity of lower limbs	−8.41 [−13.39; −3.43] *	1.23	3.47 [−2.50; 9.44]	−1.33	−14.09 [−36.62; 8.43]
Physical function	1.66 [1.09; 2.24] **	0.06	0.02 [−0.76; 0.80]	2.43	1.50 [0.18; 2.81] *
Fatigue	6.37 [3.23; 9.51] *	−0.51	−1.11 [−5.75; 3.52]	1.50	3.31 [−1.46; 8.09]
Fatigue—mental component	2.63 [1.02; 4.25] *	−1.10	−1.0 [−2.92; 0.92]	0.88	0.97 [−1.37; 3.32]
Fatigue—physical component	3.73 [1.13; 6.33] *	−0.08	−0.11 [−3.22; 2.98]	1.78	2.34 [−0.51; 5.21]

t: t-student statistic; MD: mean difference; M: mean; 95%CI: 95% confidence interval. ** Significant difference between improvements in the study groups (*p* < 0.01) * Significant difference between improvements in the study groups (*p* < 0.05).

**Table 4:** Repeated-measures analysis results of the study.

Variables	Time Effect		Sequence Effect		Time*Sequence Interaction	
	F	ES	F	ES	F	ES
Ankle pain	4.59 *	0.23	7.24 *	0.32	29.12 **	0.66
Knee pain	7.04 *	0.32	1.41	0.09	37.41 **	0.71
Functional capacity of lower limbs	6.15 *	0.29	0.96	0.06	16.26 **	0.52
Physical function	9.98 **	0.40	27.17 **	0.64	28.84 **	0.65
Fatigue	15.86 **	0.51	1.69	0.10	57.82 **	0.79
Fatigue—mental component	8.22 *	0.35	0.003	0.00	23.59 **	0.61
Fatigue—physical component	13.76 **	0.47	3.10	0.17	28.43 **	0.65

F: Fisher–Snedecor statistic; ES: effect size (η^2^_p_: partial eta squared). ** Significant difference between improvements in the study groups (*p* < 0.01) * Significant difference between improvements in the study groups (*p* < 0.05).

**Table 5:** Mean differences between the intervention and control, with 95% confidence intervals.

Variables	MD (SE)	95%CI
Ankle pain	−2.70 (0.61)	−3.98; −1.41
Knee pain	−3.11 (0.69)	−4.58; −1.63
Functional capacity of lower limbs	5.76 (1.46)	2.67; 8.86
Physical function	−0.69 (0.19)	−1.09; −0.28
Fatigue	−5.41 (0.95)	−7.44; −3.37
Fatigue—mental component	−2.00 (0.45)	−2.96; −1.03
Fatigue—physical component	−3.41 (0.64)	−4.78; −2.03

MD: mean difference; SE: Standard Error; 95%CI: 95% confidence interval.

## Data Availability

The data that support the findings of this study are available on request from the corresponding author.

## Appendix A

**Intervention**	**Technique**	**Patient** **Position**	**Physiotherapist Position**	**Action**		**Time (min)**
Myofascial techniques	Myofascial induction of hamstrings	Prone position	Stand on the side to be treated	The physiotherapist’s hands are crossed over the sciatic tuberosity and on the lower third of the posterior aspect of the thigh.		5–6
	Fascial induction of the triceps surae	Prone position with knees flexed at 90º	Stand on the side to be treated	Pressure is applied from the center toward the outside of the calves. Fingers 2–5 of both hands exert force from the junction of the gastrocnemius muscles toward the medial and lateral sides (on the medial and lateral gastrocnemius muscles, respectively). The thumbs act as “control flags” for the symmetry of the technique.		5
	Patellar myofascial release	Supine position	Stand on the side to be treated	Cranial hand on the patella (lower third of the rectus femoris), applying pressure in a cranial direction. Caudal hand in the popliteal fossa, applying traction in a caudal direction.		5–6
	Technique	Starting position		Initial action	Final action	
Proprioceptive neuromuscular facilitation	Diagonal 1	Proximal hand of the physical therapist on the distal third of the thigh, above the knee. Distal hand on the back of the foot.		Movement toward flexion, abduction, and internal rotation of the hip with eversion, dorsal flexion, and extension of the fingers.	Movement toward hip extension, adduction, and external rotation with plantar flexion and finger flexion.	
	Diagonal 2	Proximal hand on the back of the foot. Distal hand on the distal third of the thigh, above the knee.		Movement toward abduction, extension, and internal rotation with plantar flexion and flexion of the fingers.	Movement toward adduction, flexion, and external rotation with dorsiflexion and finger extension.	
